# Spidroin profiling of cribellate spiders provides insight into the evolution of spider prey capture strategies

**DOI:** 10.1038/s41598-020-72888-6

**Published:** 2020-09-24

**Authors:** Nobuaki Kono, Hiroyuki Nakamura, Masaru Mori, Masaru Tomita, Kazuharu Arakawa

**Affiliations:** 1 Institute for Advanced Biosciences, Keio University, 403-1 Nipponkoku, Daihouji, Tsuruoka, Yamagata 997-0017 Japan; 2 Spiber Inc., 234-1 Mizukami, Kakuganji, Tsuruoka, Yamagata 997-0052 Japan

**Keywords:** Molecular evolution, Proteomics, Transcriptomics

## Abstract

Orb-weaving spiders have two main methods of prey capture: cribellate spiders use dry, sticky capture threads, and ecribellate spiders use viscid glue droplets. Predation behaviour is a major evolutionary driving force, and it is important on spider phylogeny whether the cribellate and ecribellate spiders each evolved the orb architecture independently or both strategies were derived from an ancient orb web. These hypotheses have been discussed based on behavioural and morphological characteristics, with little discussion on this subject from the perspective of molecular materials of orb web, since there is little information about cribellate spider-associated spidroin genes. Here, we present in detail a spidroin catalogue of six uloborid species of cribellate orb-weaving spiders, including cribellate and pseudoflagelliform spidroins, with transcriptome assembly complemented with long read sequencing, where silk composition is confirmed by proteomics. Comparative analysis across families (Araneidae and Uloboridae) shows that the gene architecture, repetitive domains, and amino acid frequencies of the orb web constituting silk proteins are similar among orb-weaving spiders regardless of the prey capture strategy. Notably, the fact that there is a difference only in the prey capture thread proteins strongly supports the monophyletic origin of the orb web.

## Introduction

An orb-weaving spider generally has morphologically distinct types of abdominal silk glands, and each silk gland synthesizes specialized silk proteins (spidroins) and produces up to seven different silks with diverse mechanical properties^1–4^. These varied mechanical properties enable spiders to utilize silks in several situations, including egg protection, dispersal, locomotion, and prey capture^5,6^. In particular, the evolution of prey capture threads has had a strong effect on spider diversity^3,4,7,8^.

Capture threads can be categorized into glue or mechanical stickiness. Almost all orb-weaving spiders use adhesive capture threads coated with viscid glue. Viscid glue is an aqueous droplet produced in an aggregate gland, with flagelliform silk used for the core fibre^3,9–13^. The aggregate gland glue is composed of adhesive glycoproteins surrounded by an aqueous solution of low molecular mass compounds (LMMCs)^14^. Therefore, the difference in droplet size changes the proportion of water and achieves different viscosity^15^. Spiders that use a dry capture thread with mechanical stickiness instead of the viscid capture silk are called cribellate spiders. A cribellate spider spins the mechanically sticky silk produced by cribellar spidroin (CrSp) from a cribellum in the plate-like spinning organ. The cribellate silk is generated by a small acinous-shaped cribellate silk gland^16,17^. Typical cribellate spiders, such as deinopids and uloborids, use a pseudoflagelliform silk produced by pseudoflagelliform spidroin (Pflag) instead of flagelliform silk produced by flagelliform spidroin (Flag) for core fibres^18–20^, which are coated with finely brushed cribellate silks^17,21,22^ as is found in ecribellate orb-weavers. The cribellate capture threads are composed of materials with different mechanical properties, the core fibre is stiff, and the cribellate nanofibrils is extensible^23^. This composite has a synergistic mechanical function that the core fibres are broken intermittently at the early stage of tensile deformation, and the threads gradually unravel and elongate to increase thread extension^24^. The cribellate capture threads are dry and, rather than adhering to prey, entangle prey^25–27^. The stickiness of cribellar fibre is achieved through interactions with the epicuticular waxes of insects^28^. The cribellate spider *Progradungula otwayensis* (family Gradungulidae) varies the spinning behaviour of cribellate silk and produces various prey capture threads with different mechanical properties^29^. It is thought that viscid threads produced by ecribellate orb-weavers are coated with aqueous droplets of the aggregate gland glue and are less expensive to produce than cribellate capture silk^9,30–34^. On the other hand, although it takes a long time to build a cribellate orb web, these webs are long lasting^35^.

The transition from dry cribellate capture silk to viscid silk is difficult to understand because they require different conditions to function. Thus, the fundamental differences might indicate that cribellate and ecribellate spiders have evolved each orb architecture independently^36^. On the other hand, the shared synapomorphy of web architecture and building sequences suggests a monophyletic origin of the orb web (ancient orb web hypothesis)^37–40^. This ancient orb web hypothesis has been discussed based on a molecular phylogeny with high resolution across broad taxonomic scale. This hypothesis is supported when combined with morphological and behavioral characters^41^, but is rejected from pure transcriptomic phylogenomic analysis^42^.

To understand the origin of the orb web, we need to look not only at the morphological and behavioural traits but also at molecular information of spidroin material that makes-up the orb-web. However, contrary to the ecribellate spiders, there is still little molecular information about the spidroins in cribellate spiders. Regarding the *CrSp* gene, only partial sequences of candidate genes in *Tengella perfuga* (family Zoropsidae) and *Stegodyphus mimosarum* (family Eresidae, KFM60634.1) have been reported^43^, and the *Pflag* gene sequence is also only known in *Deinopis spinosa* (family Deinopidae)^44^.

Here, we prepare the spidroin data in cribellate orb-weaving spider (family Uloboridae) with multiple omics approaches and conduct phylogenetic analysis. The Uloboridae cribellate spider family is an appropriate target for this study. Uloborids are cosmopolitan cribellate spiders belonging to 18 genera (world spider catalog: https://wsc.nmbe.ch/), and the general eastern Asian uloborid is in the genus *Octonoba*^45^. Using six uloborid species (*Octonoba sybotides*, *O. yesoensis*, *O. grandiprojecta*, *O. varians*, *O. grandiconcava*, and *O. okinawensis*), we curate the spidroin data set with transcriptome assembly complemented with long read sequencing, where silk composition is confirmed by proteomics. The cribellate data set provides the insight into the evolution of the spider web.

## Results

### *Octonoba* spider cDNA/gDNA sequencing and assembly

De novo transcriptome analyses were performed using cDNA-seq with six species of uloborid spiders (*O. sybotides*, *O. yesoensis*, *O. grandiprojecta*, *O. varians*, *O. grandiconcava*, and *O. okinawensis*) and provided transcriptome references. Total RNA was extracted from each whole body, and synthesized cDNA was sequenced with an Illumina sequencer. 150-bp paired-end sequencing produced a total of 37 M reads on average that passed the quality filter (Table 1). These reads were assembled by each sample, and an average of 41,795 contigs (N50 ranging from 1914 to 2282 bp) were obtained (Table 1). The quality of the assembled gene set was estimated by the BUSCO completeness score, and the completeness score of the Arthropoda gene model ranged from 71.29 to 93.15%. In addition to the transcriptome references, we performed long-read sequencing of genomic DNA (gDNA) with a nanopore sequencer. Spidroin genes are very long and almost entirely composed of the highly repetitive domain between nonrepetitive N/C-terminal domains^3,12,46^. Therefore, short-read sequencing alone is not sufficient, and hybrid sequencing combined with long-read sequencing is necessary to identify such genes^47,48^. The gDNA nanopore sequencing produced 133,525 reads with a total of 1.86 G nucleotides on average (Table S1).

**Table 1:** Transcriptome assembly statistics for *O. yesoensis*, *O. sybotides*, *O. okinawensis*, *O. varians*, *O. grandiconcava*, and *O. grandiprojecta*.

Transcriptome	*Octonoba yesoensis*	*Octonoba sybotides*	*Octonoba okinawensis*	*Octonoba varians*	*Octonoba grandiconcava*	*Octonoba grandiprojecta*	Average
Scaffold number	44,188	39,956	38,452	41,438	46,760	39,974	41,795
Total scaffold length (bp)	63,318,496	52,083,274	36,914,372	57,234,666	70,811,127	53,312,272	55,612,368
Average scaffold length (bp)	1432	1303	960	1381	1514	1333	1321
Longest scaffold (bp)	13,479	13,833	9029	16,015	13,033	10,670	12,677
Shortest scaffold length (bp)	196	201	199	178	201	129	184
N50 (bp) (# of scaffolds in N50)	2051 (#10,031)	1913 (#8798)	1428 (#8180)	2046 (#9170)	2282 (#10,409)	1967 (#8724)	1948 (#9219)
N90 (bp) (# of scaffolds in N90)	712 (#30,019)	630 (#27,000)	438 (#26,495)	682 (#27,511)	745 (#30,953)	652 (#26,792)	643 (#27,937)
BUSCO completeness (%)	93.15	89.68	71.29	93.43	88.74	92.50	88.13
Reads	41,728,820	29,673,788	35,015,922	43,196,434	29,637,102	44,532,118	37,297,364
Location	Japan:Yamagata	Japan:Kumamoto	Japan:Okinawa	Japan:Kumamoto	Japan:Kagoshima	Japan:Okinawa	
DRR	DRR155974	DRR156101	DRR155866	DRR156045	DRR157004	DRR157173	
Sampling date	11-Jun-15	11-May-15	21-Nov-15	11-May-15	21-Mar-17	7-Nov-16	

### Spidroin gene catalog for cribellate spiders

**Figure 1:**
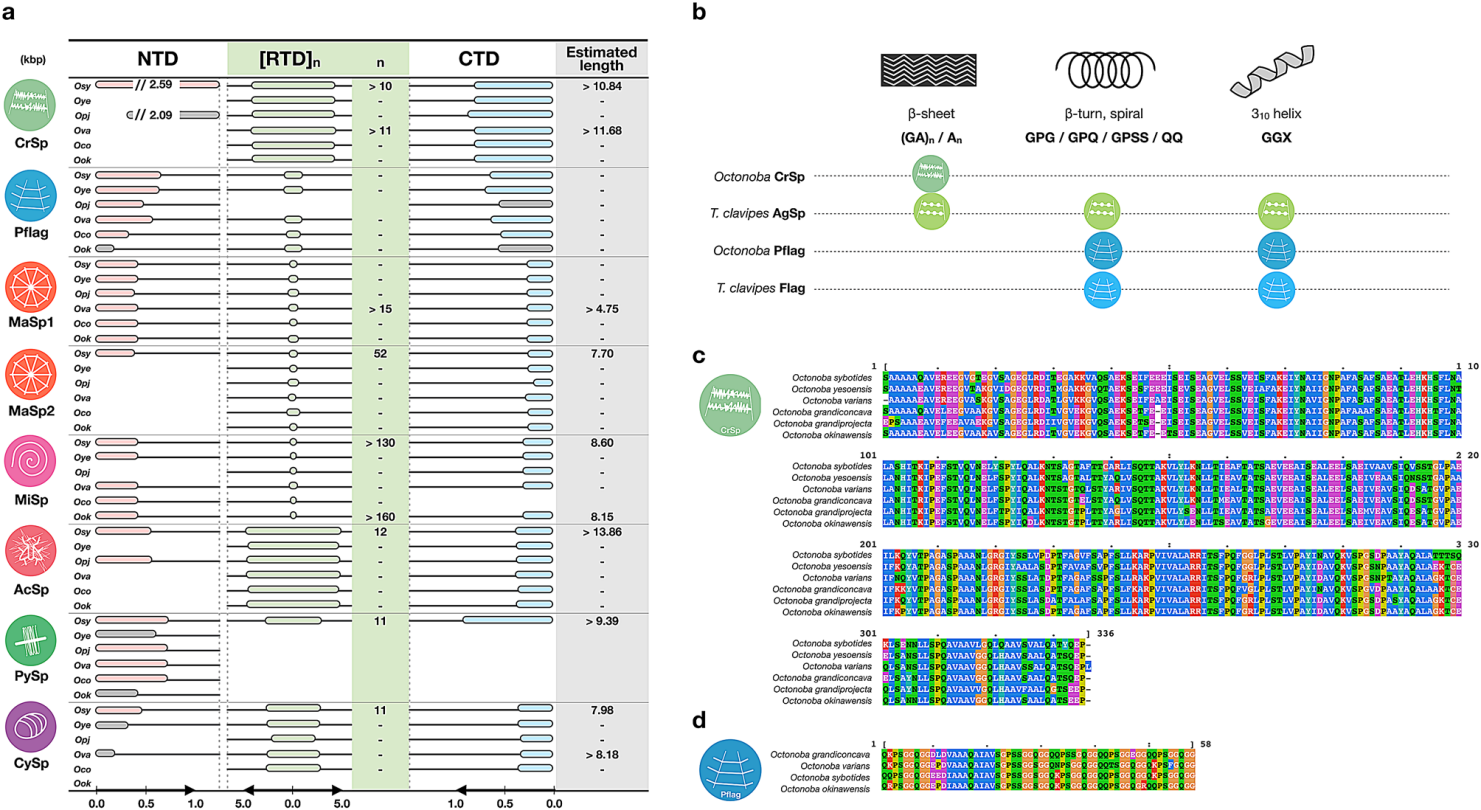
Overview of spidroin catalog of six *Octonoba* spiders. **a** Spidroin genic architectures obtained from the genome and transcriptome data. The eight circular icons represent spidroin types. The lengths of the NTD (N-terminal domain) and CTD (C-terminal domain) are represented by the red and blue bar lengths, respectively, and grey bars indicate partial domains. The average length of each RTD (repetitive domain) is represented by a green bar, and the estimated iteration number is described in column “n”. **b** Motifs of CrSp and Pflag in *Octonoba* spiders and AgSp and Flag in *Trichonephila clavipes*. **c** and **d** Alignment results of the typical repetitive domains of CrSp and Pflag.

Using assembled transcriptome scaffolds and long reads of gDNA, the spidroin gene sequences were curated, and the whole spidroin gene catalogue of cribellate spiders was produced (Fig. 1a). The full or partial-length gene sequences of all known spidroin types, such as a web radii and drag line silk sequence: major ampullate spidroin (MaSp), a web reinforcement sequence: minor ampullate spidroin (MiSp), a prey-wrapping silk sequence: aciniform spidroin (AcSp), an attachment silk protein sequence: pyriform spidroin (PySp), and an egg case silk sequence: cylindrical spidroin (CySp, also known as TuSp for tubuliform spidroin), were obtained. The lengths of some genes were estimated based on the long reads. Almost all spidroin genes were approximately 10 kbp in length, a size that is common to the spidroin genes in Araneoid spiders^48,49^. Complete full-length sequences were obtained for *MaSp2*, *CySp*, *PySp*, and *MiSp* (Fig. S1). To eliminate the possibility of chimeric artefacts, the continuity of each spidroin gene was validated by read mapping, and it was confirmed that the N/C-terminal regions were one continuous molecule (Fig. S2).

The cribellate spider-associated genes (*CrSp* and *Pflag*) were also searched based on the partial *CrSp* candidate sequence in *T. perfuga*^43^ and the Flag sequence in *Araneus ventricosus*^48^. As a result, a full picture of the genes, including the repetitive and N/C-terminal domains of *CrSp* and *Pflag*, was obtained (Fig. 1a). The lengths of the *CrSp* C-terminal and repetitive domains were 962 bp and 1009 bp on average, respectively. The repetitive domains were relatively long, similar to the architecture of *AcSp*, which has a repetitive domain with an average length of 1124 bp. The start codon of the *CrSp* N-terminal domain was determined based on the location of the signal peptide and Kozak rule (Fig. S3). The N-terminal length of other spidroins averages 500 bp, whereas the CrSp N-terminal domain was very long, more than 2 kbp (Fig. 1a). Because this long N-terminal domain was also found in another cribellate spider, *S. mimosarum* (family Eresidae) (KFM70693.1, Fig. S4), it was confirmed that the *CrSp* sequence is widely conserved among cribellate spiders. *CrSp* had a repetitive domain composed of at least 11 tandem copies and no intronic regions. The iteration number of repetitive domains was estimated using the long reads obtained by gDNA sequencing (Fig. S5). On the other hand, the repetitive domains of *Pflag* were approximately 200 bp in length, which was shorter than the repetitive domains of *Pflag* in *D. spinosa*^44^ (Fig. S6). Our spidroin catalog also revealed the motif variety in the repetitive domain of spidroins associated with prey capture threads. In the *CrSp* repetitive domain, a β-sheet motif (A_n_), a known characteristic motif of MaSp, was found, and this feature was similar to the viscid glue protein (AgSp: aggregate spidroin) in *T. clavipes* (Fig. 1b,c). However, as mentioned in a previous study^43^, other than that, only features commonly found in spacers were observed (TT or SS), and no other noteworthy motif was found. On the other hand, Pflag had similar motifs as Flag, although the motif lengths of Pflag and Flag were different. In addition to the GGX motif reported in the Flag repetitive domain, GPSS and QQ motifs were found in Pflag (Fig. 1d).

### Multiple omics profiling for CrSp and Pflag

**Figure 2:**
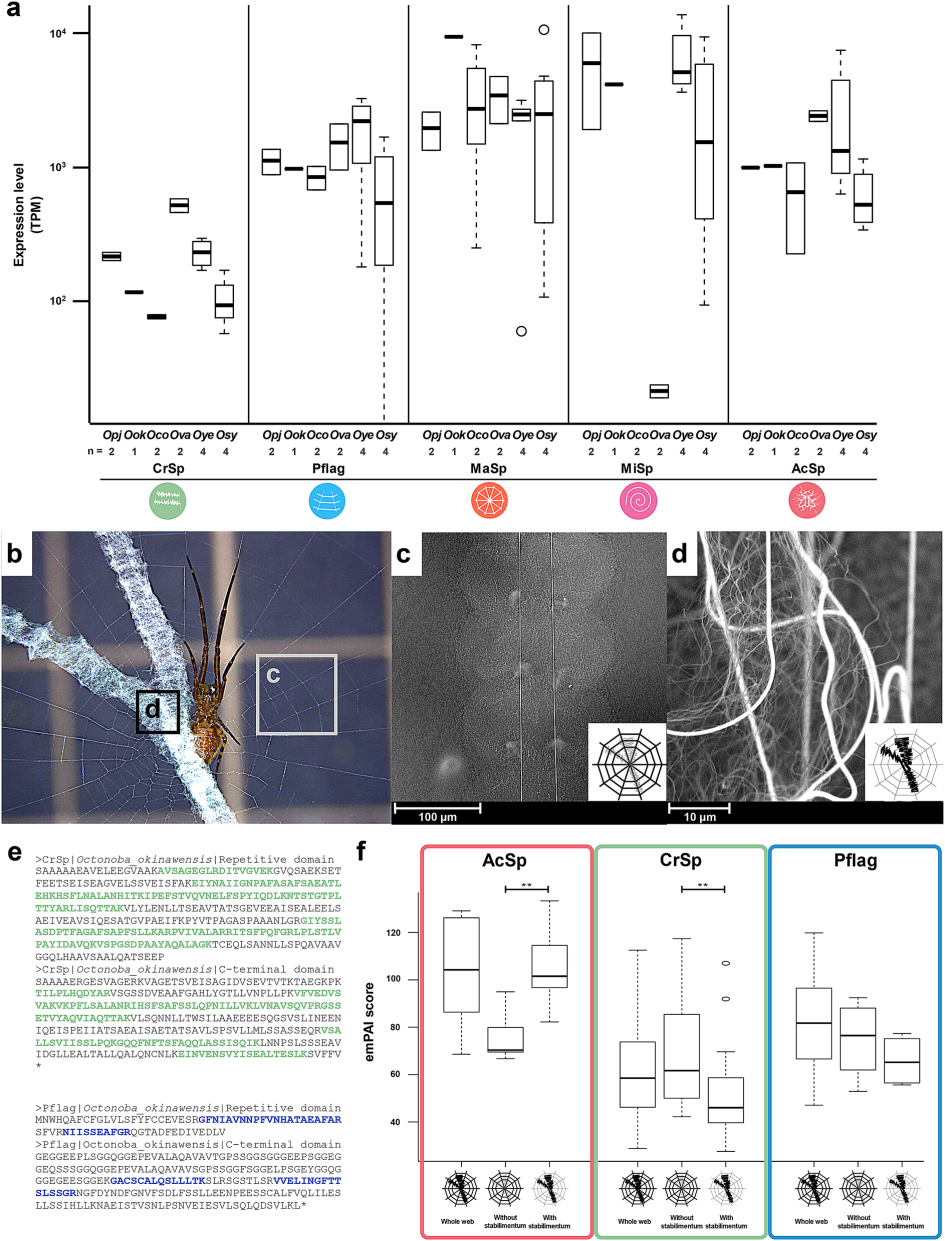
Expression and proteome analysis of *Octonoba* web threads. (**a**) Gene expression levels of spidroin genes in the whole body of six *Octonoba* spiders (Oco: *O. grandiconcava*; Ova: *O. varians*; Ook: *O. okinawensis*; Opj: *O. grandiprojecta*; Oye: *O. yesoensis*; Osy: *O. sybotides*). (**b**) An *Octonoba varians* web with the radius and spiral area, including cribellate silk (SEM image **c**) and stabilimentum (SEM image **d**). **e** The coloured amino acids in the CrSp and Pflag protein sequences represent the peptides detected from web threads by mass spectrometry analysis. **f** emPAI scores of spidroins in each web part (whole web, area without stabilimentum, and stabilimentum area).

*CrSp* and *Pflag* gene sequences obtained from genome data by homology search with known sequences are guaranteed by the sequence similarity only. Whether they worked in vivo was confirmed by expression profiling of the transcriptome and proteome. Transcriptome analysis was performed on cDNA sequencing samples from six species of *Octonoba* spiders. cDNA was synthesized from mRNA extracted from whole body total RNA, and two or more biological replicates were prepared for each species, except for *O. okinawensis*. According to the profile results, all spidroin genes were expressed in all spider bodies (Fig. 2a). Even relatively lowly expressed *CrSp* showed an expression level of approximately 100 TPM, with an overall trend toward higher expression of MaSp and MiSp observed. Moreover, a slight correlation relationship of gene expression patterns was observed between *CrSp* and *Pflag* (Fig. S7).

The contributions of CrSp and Pflag proteins to the orb web composition were investigated by proteome analysis. According to a previous study, in the cribellate orb web, the cribellate silks are organized as a mat, are curled and enclose the spiral Pflag axial fibres with a puffy structure^50^. In addition, uloborid spiders decorate their orb web to reflect UV light and catch the prey insects attracted by the UV light^45^. This decoration is called stabilimentum and consists of AcSp^4^. To confirm the presence of CrSp and Pflag, proteome analysis was performed separately for each web part, such as the radius and spiral area, the stabilimentum area, and the whole web (Fig. 2b–d). We expected that the radius and spiral area would mainly include MaSp, MiSp, CrSp, and Pflag and that the stabilimentum area would consist of AcSp. The cribellate orb web sample was collected from the orb web built by *O. okinawensis* in a laboratory cage. The proteome analysis confirmed the presence of main spidroins, such as MaSp, MiSp, CrSp, Pflag, and AcSp, in the cribellate spider’s web (Fig. S8). The CrSp and Pflag sequences obtained from the genome were also observed as peptide fragments in the web (Fig. 2e). The quantification of the protein amount was calculated as an emPAI (exponentially modified protein abundance index; Fig. 2f and Table S2)^51^. The AcSp amount in the stabilimentum area was significantly higher than that in the radius and spiral area, and AcSp was shown to be the primary protein of the stabilimentum. On the other hand, for the radius area where CrSp was used as the coating thread, high amounts of CrSp and Pflag were detected. In particular, CrSp was used significantly more in the radius than in the stabilimentum. According to the multiple omics analysis from genomic DNA, RNA, and peptide sequences, we validated the newly found CrSp and Pflag.

### Phylogenetic analysis of spidroin genes

**Figure 3:**
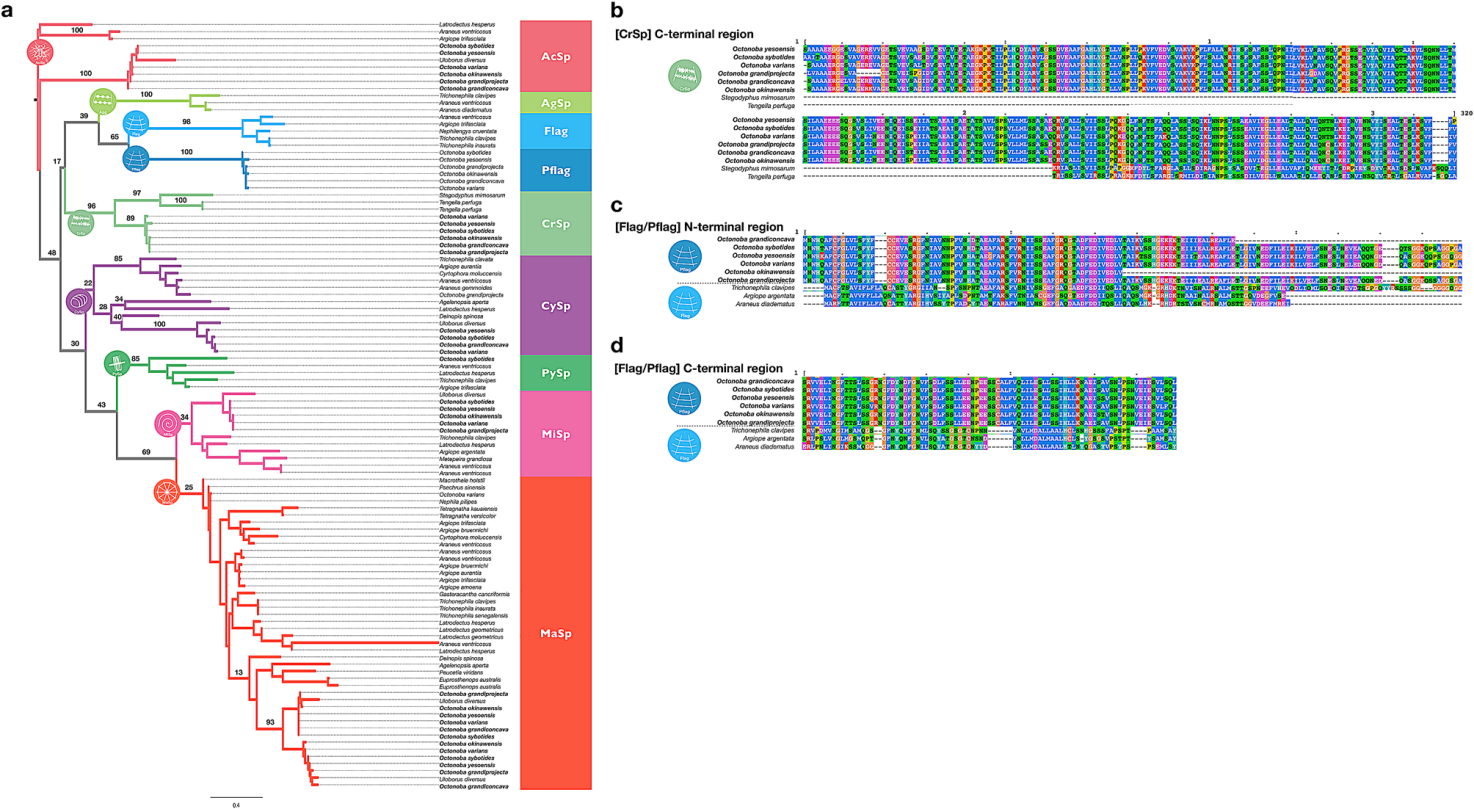
Spidroin phylogeny. (**a**) Phylogenetic analysis of all spidroin C-terminal regions. Each spidroin type is coloured, and *Octonoba* spiders are indicated by bold font. Branch labels designate bootstrap support values. All spidroin data are listed in Tables S2 and S3. Alignment results of the CrSp C-terminal domain (**b**), Flag and Pflag N-terminal domain (**c**) and C-terminal domain (**d**).

**Figure 4:**
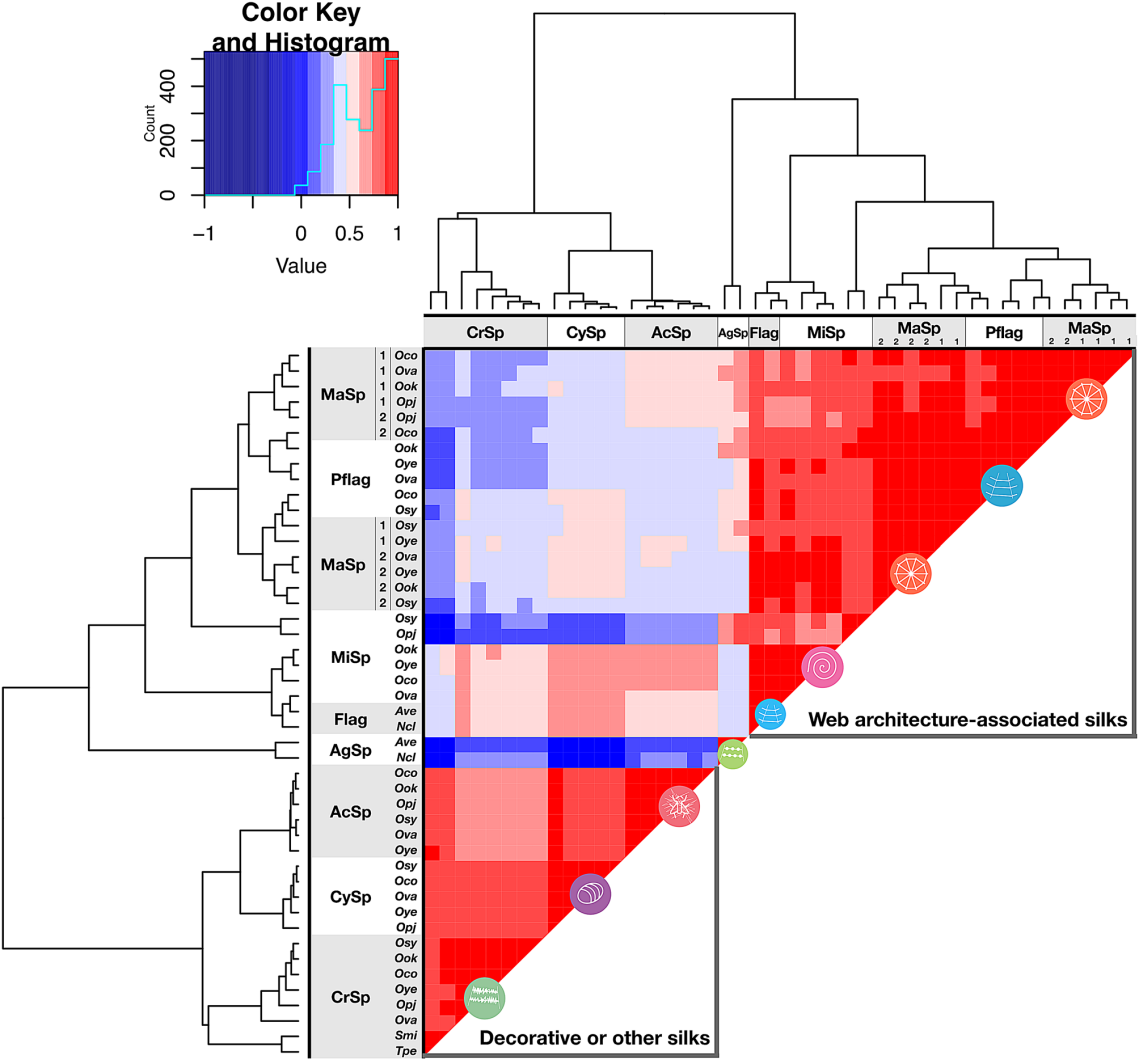
Hierarchical clustering of spidroins based on amino acid composition in *Octonoba* spidroin. Hierarchical clustering based on amino acid in repetitive domains of spidroins. Oco: *O. grandiconcava*; Ova: *O. varians*; Ook: *O. okinawensis*; Opj: *O. grandiprojecta*; Oye: *O. yesoensis*; Osy: *O. sybotides*; Ave: *A. ventricosus*; Ncl: *T. clavipes*; Smi: *S. mimosarum*; Tpe: *T. perfuga.*

Using our *Octonoba* spidroin catalog evaluated by multiple omics analysis, we compared the N/C-terminal regions with known spidroin genes in other spiders. The known spidroin gene sequences were obtained from a public database (NCBI) without limiting the taxonomic families (Tables S3 and S4). Based on the N/C-terminal regions, each of nine spidroin genes (*MaSp*, *MiSp*, *AcSp*, *CySp*, *PySp*, *AgSp*, *Flag*, *Pflag*, and *CrSp*) belonged to a monophyletic group regardless of the species taxonomy (Figs. 3a and S9). The C-terminal regions of CrSps were classified into a group consisting of previously reported CrSp candidates in *T. perfuga* and *S. mimosarum*^43^. Although Pflags form an independent group without Flags in the phylogenetic tree (Figs. 3a), the alignment analysis showed that the terminal regions are relatively well conserved, and Pflag and Flags seemed to share a monophyletic origin (Fig. 3c,d). On the other hand, since the AgSp clade includes the Flag/Pflag clade, it suggested that the CrSp and AgSp are not derived from a monophyletic lineage. This tendency was also observed for the amino acid compositions. Figure 4 shows the correlation matrix heatmap of amino acid compositions in the repetitive domains of all *Octonoba* spidroins. Intriguingly, the *Octonoba* spidroins were separated into two groups: web architecture-associated silks (MaSp, MiSp, and Pflag) and decorative or other silks (CrSp, AcSp, and CySp). Note that the AgSp in *T. clavipes* and *A. ventricosus* showed a unique amino acid composition different from that indicated by both groups.

## Discussion

Our study clarifies the molecular background of cribellate spiders and may enable us to perform large-scale phylogenetic analysis of spidroin variety evolution. So far, the whole spidroin catalog in orb-weaving spider has been reported for *T. clavipes*, *A. ventricosus*, and *Caerostris darwini*^48,49,52^, and these species are ecribellate orb-weaving spiders belonging to the superfamily Araneoidea. This study targets six spider species that are cribellate orb-weaving spiders belonging to the family Uloboridae and are distantly related to the superfamily Araneoidea. Spidroin catalogues were curated from each of the six species of uloborid spiders. This curation was realized using a previously reported method combining hybrid sequencing and the SMoC algorithm (Spidroin Motif Collection)^48,49^. Since it is impossible to sequence the heavily repetitive domains in spidroin using a short read sequencer^53,54^, the hybrid approach with short and long read sequencing is essential. Contig extension with an extremely large k-mer based on the short and long reads provided almost all spidroins. The detailed procedures are described in the “Methods” section.

The *Octonoba* spidroin catalogue including *CrSp* and *Pflag* genes was confirmed by multiple omics approaches, such as genomics, transcriptomics, and proteomics. Previously, partial spidroin genes of cribellate spiders have been found in *O. varians*, *U. diversus* (family Uloboridae), *T. perfuga* (family Zoropsidae), *S. mimosarum* (family Eresidae), and *Deinopis spinosa* (family Deinopidae)^43,44,55^. In *O. varians*, three approximately 300 aa long segments annotated as the “dragline silk spidroin 1 gene” have been reported (AY666057, AY666058, and AY666059)^55^. Those sequences matched to the C-terminal region of *MaSp* in our catalog (Fig. S10). The first CrSp gene sequence was reported by Correa-Garhwal and colleagues in *T. perfuga* (Zoropsidae; Dictynoidea). According to the alignment with previously reported genes, the C-terminal region of CrSp was conserved among the cribellate spiders (*Octonoba*, *T. perfuga* and *S. mimosarum*, Fig. 3b). In the case of the repetitive domain, although the similarity was not evaluated quantitatively since the reported domains of *T. perfuga* and *S. mimosarum* were too short, conservation was observed (Fig. S11).

Each spidroin was very well conserved among species in the genus *Octonoba* with respect to the spidroin gene sequence, architecture, and amino acid composition (Figs. 3 and 4). Moreover, except for genes related to prey capture threads (CrSp and AgSp), the spidroin genes in cribellate spiders showed very standard sequence features for orb-weaving spiders and did not differ from the sequence features of ecribellate spiders. Clustering analysis based on spidroin gene sequences showed the clear categorization of spidroin families (Fig. 3). Although Pflag and Flag were not extremely similar (Fig. 3c,d), taking a global phylogenetic view of spidroin, since Flag and Pflag seem to be categorized into the same clade, they may have a monophyletic origin. On the other hand, CrSp and AgSp were individually clustered, and their sequences were different from each other. The motifs and amino acid compositions in the repetitive domain varied completely (Figs. 4 and S11), and computationally predicted protein structures were different from each other (Fig. S12). It is known that the variety of mechanical properties in spider silk is realized by the secondary structure of protein designated by each motif in repetitive domain^40^. The difference in the secondary structure of CrSp and AgSp shown in Fig. S12 suggests that their mechanical properties are different.

The phylogenetic tree visualizes the evolutionary relationship at various scales and helps us to understand the relationship intuitively. However, the arguments based on the tree must be very cautious. Recently, in opposition to the conventional “ancient orb web hypothesis” that orb-weaving spiders have a single origin^37–39,56^, Fernández and colleagues argued that the orb-weaving spiders have at least three independent origins (Araneoidea, Deinopidae, and Uloboridae) and the prey capture webs evolved multiple times based on genomic-scale data^42^. They analysed lineage diversification and found that the ANE (superfamily Araneoidea, Nicodamoidea, and Eresoidea) and the UR (family Uloboridae, Deinopidae, Oecobiidae, Hersiliidae, and RTA clade) grades appeared 200–250 million years ago^42^. In these two grades, only Araneoidea, Hersiliidae, and RTA are ecribellate, and only Uloboridae and a part of Araneoidea construct orb webs. The cribellate and orb-weaving spiders were mixed in each grade. However, the following year, that hypothesis was again challenged. This phylogenetic analysis was thoroughly reviewed by Coddington and colleagues through the selection of appropriate algorithms and rigorous data curation, and it is shown that the spiders have been lost the web 5–7 times, not gained multiple times^57^. In other words, it was reconfirmed that the single origin was the most likely explanation. Our results based on the spidroin sequences also rejected the convergent evolution of the orb-web and strongly supported the “ancient orb web hypothesis”. The uloborid spidroin catalogue revealed that the cribellate spidroin sequences are well conserved among the cribellate spiders (Fig. S4), and the orb-web-associated spidroin sequences are also conserved among orb-weaving spiders (Fig. 3). Sequence conservation dismisses the possibility of convergent evolution and strongly supports the existence of a common ancestor harbouring cribellate orb webs like uloborid spiders. AgSp has been reported not only from the orb-weaving spiders, but also from Theridiidae spiders that build a cob web^54,58–61^. Hence, our molecular analyses suggest the possibility that complex ANE and UR grades appear as a result of the simple evolutionary history of CrSp or orb web losses or AgSp gains. It was suggested that the acquisition of the prey capture strategy and the web structure might be independent.

## Methods

### Spider sample preparation

Uloborid spider specimens (*O. sybotides*, *O. yesoensis*, *O. grandiprojecta*, *O. varians*, *O. grandiconcava*, and *O. okinawensis*) were collected from Yamagata, Hokkaido, Kanagawa, Kumamoto, Wakayama, Okinawa, and Kagoshima Prefecture, Japan (from May 2015 to December 2018). The samples were stored in a plastic container (PAPM340, RISUPACK CO., LTD.) and transported live back to the laboratory. The collected spiders were initially identified based on morphological characteristics, and identifications were confirmed using the cytochrome c oxidase subunit 1 (*COX1*) sequence in the Barcode of Life Data System (BOLD: https://www.barcordinglife.org). Based on previously established field sampling protocol^62^, immediately upon arrival in the laboratory, uloborid spiders were immersed in liquid nitrogen (LN2) for cDNA or genome sequencing and stored at − 80 °C. The SEM images of silk were taken with a scanning electron microscopy Phenom World model Phenom Pro-X using a web built in the laboratory.

### Total RNA extraction

Total RNA was extracted from 15 specimens of six uloborid spider species (*O. sybotides*, *O. yesoensis*, *O. grandiprojecta*, *O. varians*, *O. grandiconcava*, and *O. okinawensis*) based on the spider transcriptome protocol^62^. We immersed the flash frozen specimens into 1 mL TRIzol Reagent (Invitrogen) before homogenizing with a metal cone using the Multi-Beads Shocker (Yasui Kikai). Using an RNeasy Plus Mini Kit (Qiagen) automated with QIACube (Qiagen), the extracted RNA was purified. The total RNA was qualified with a NanoDrop 2000 (Thermo Scientific) and TapeStation 2200 with RNA Screen Tape (Agilent Technologies). The quantity was measured with Qubit Broad Range (BR) RNA assay (Life Technologies).

### HMW (high molecular weight) gDNA extraction

According to a previous report^48^, HMW gDNA was extracted from the whole bodies of flash frozen spiders using Genomic-tip 20/G (QIAGEN). The specimens were quickly homogenized using BioMasher II (Funakoshi) and mixed with 2 mL of Buffer G2 (QIAGEN), including 200 µg/mL RNase A. After the addition of 50 µL of proteinase K (20 mg/mL), the lysate was incubated at 50 °C for 12 h on a shaker (300 rpm). The lysate was centrifuged at 5,000 × g for 5 min at 4 °C to pellet the debris, and the aqueous phase was loaded onto a pre-equilibrated QIAGEN Genomic-tip 20/G (QIAGEN) by gravity flow. The QIAGEN Genomic-tip 20/G (QIAGEN) was then washed three times, and the DNA was eluted with high-salt buffer (Buffer QF) (QIAGEN). The eluted DNA was desalted and concentrated by isopropanol precipitation and resuspended in 10 mM Tris–HCl (pH 8.5). Extracted gDNA was qualified using a NanoDrop 2000 (Thermo Scientific) and TapeStation 2200 with genomic DNA Screen Tape (Agilent Technologies) and quantified using a Qubit Broad Range (BR) dsDNA assay (Life Technologies).

### Library preparation and sequencing of cDNA and gDNA

Library preparation was performed using the same protocol as the previously conducted for cDNA/gDNA sequencing of *A. ventricosus* and *E. variegata*^47,48^. The libraries for cDNA sequencing were prepared using the NEBNext Ultra RNA Library Prep Kit for Illumina (New England BioLabs) according to the manufacture’s protocol. Using NEBNext Oligo d(T)25 beads without a wash step, mRNA was isolated from 100 µg of extracted total RNA. The cDNA synthesis was performed using ProtoScript II Reverse Transcriptase and NEBNext Second Strand Synthesis Enzyme Mix. Synthesized double-stranded cDNA was ligated with a NEBNext Adaptor for Illumina after end repair using NEBNext End Prep Enzyme Mix. After USER enzyme treatment, cDNA amplification by PCR was performed with the following conditions: 20 μL cDNA, 2.5 μL index primer, 2.5 μL universal PCR primer, 25 μL NEBNext Q5 Hot Start HiFi PCR Master Mix 2X; 98 °C for 30 s and 12 cycles each of 98 °C for 10 s, 65 °C for 75 s and 65 °C for 5 min. In the case of nanopore sequencing of gDNA long reads, we purified HMW gDNA by > 8 kb size selection using a BluePippin system (Sage Science) with a 0.75% agarose gel cassette before starting the library preparation with the 1D library protocol (SQK-LSK108, Oxford Nanopore Technologies). The cDNA sequencing was performed with a NextSeq 500 (Illumina, Inc.) using 150 bp paired-end reads with a NextSeq 500 High Output Kit (300 cycles). Sequenced read quality was assessed with FastQC (v0.10.1: https://www.bioinformatics.bbsrc.ac.uk/projects/fastqc/). Nanopore sequencing of gDNA long reads was performed with v9.4 SpotON flow cells (FLO-MIN106) on a GridION instrument (Oxford Nanopore Technologies).

### De novo transcriptome assembly

The de novo assembly was performed using Bridger [r2014‐12‐01:^63^] with the following options: pair_gap_length = 0 and k‐mer = 31. The transcriptome assembly was validated by analysis of BUSCO (Benchmarking Universal Single-Copy Ortholog) completeness with the Arthropoda lineage gene set ^64^.

### Spidroin gene curation

*Octonoba* spidroin gene curation was performed based on a previously reported SMoC (Spidroin Motif Collection) algorithm^48^. This algorithm was implemented using the hybrid assembly with short and long reads. Illumina short reads were assembled in contigs by the de Bruijn graph method, and a BLAST search for N/C-terminus and repetitive region candidates was carried out on the contigs. The obtained candidates were used as seeds for screening the short reads harbouring an exact match of extremely large k-mers up to the 5′-end, and the short reads were aligned on the 3′-side of the matching k-mer to build a PWM (Position Weight Matrix). Based on stringent thresholds, the seed sequences were extended until neighbouring repeats appeared. Finally, the collected full-length subsets of the repeat units were mapped onto error-corrected long reads. The data about the spidroin gene length or architecture were curated manually based on the mapped long reads.

### Phylogenetic analysis

Phylogenetic reconstructions of the N/C-terminal spidroin regions were performed by MAFFT alignment of the first 90 N-terminal amino acid residues and the last 80 C-terminal amino acid residues of *Octonoba* spiders with the corresponding amino acid residues of all other available spidroin sequences (Tables S3 and S4). Maximum likelihood gene trees were constructed with 1,000 bootstrap replicates by RAxML v8.2.11^65^. FigTree v1.4.3 (https://tree.bio.ed.ac.uk/software/figtree/) was used as a viewer for the trees.

### Expression analyses

Transcript abundances were estimated by kallisto v0.42.2.1 in transcripts per million (TPM). Each transcriptome data set was obtained from whole-body cDNA sequencing of *Octonoba* spiders, and our transcriptome assembly was used as the reference.

### Orb web thread collection and preparation for proteome analysis

Stabilimentum and other thread components were directly collected from the orb web of *Octonoba okinawensis*. All silk samples were gently washed with 100 µL of base buffer [50 mM ammonium carbonate in distilled water] with 0.1% SDS per 0.5–1.0 mg of silk at RT for 1–2 min. After the supernatant removal, silk samples were immersed in 10 µL of 6 M guanidine-HCI buffer (pH8.2) and protein extraction was performed by sonication for 10 min with Bioruptor II (BM Equipment Co., Ltd.). The lysates were reacted with DTT (dithiothreitol) for 30 min at 37 ℃ followed by IAA (iodoacetamide) for 30 min at 37 ℃ in the dark. After five times dilution by 50 mM ammonium carbonate in distilled water, the samples were reacted with Lys-C for 3 h at 37 ℃ followed by trypsin for 16 h at 37 ℃ to digest proteins. The enzymatic reaction was quenched by acidification with TFA (trifluoroacetic acid) and the digested samples were desalted using Empore SDB-XC membrane (3 M) packed StageTips.

### Liquid chromatography mass spectrometry analysis

Liquid chromatography mass spectrometry was performed using the same protocol as the previously conducted for dragline silk of *A. ventricosus*^48^. We dissolved each silk sample into 12 µL of 0.1% TFA acid and 5% acetonitrile and loaded 5 µL of the solution on a hand-made spray needle column (ACQUITY UPLC BEH C18 materials, 100 µm i.d. Dr. Maisch GmbH, Germany, 5 µm tip i.d., 150 mm length) using an HTC-PAL autosampler (CTC Analytics, Zwingen, Switzerland). Separation of peptide fragments in the samples through the column was performed by reversed-phase chromatography in linear gradient mode using an UltiMate 3000 nanoLC Pump (Dionex Co., Sunnyvale, CA, USA). Two mobile phases, (A) acetic acid/dimethyl sulfoxide/water (0.5:4:96, v/v/v) and (B) acetic acid/dimethyl sulfoxide/acetonitrile (0.5:4:96, v/v/v) were mixed at a flow rate of 500 nL/min. The composition was changed as follows: (A) + (B) = 100%, (B) 0–35% (0–60 min), 35–80% (60–65 min), 80% (65–70 min), and 0% (70.1–95 min). The peptides ionized at 2600 V by the positive electrospray method were injected into an LTQ orbitrap XL ETD (Thermo Electron, San Jose, CA, USA) and detected as peptide ions (scan range: *m/z* 400–1500; mass resolution: 60,000 at *m/z* 400). The top 10 peaks of multiply charged peptide ions were subjected to collision-induced dissociation (isolation width: 2, normalized collision energy: 35 V, activation Q: 0.25, activation time: 30 s) to identify the amino acid sequences.

### Database search for protein identification

The peak lists were created from LC–MS raw data files with msconvert.exe, which was provided by ProteoWizard^64^, and analysed with Mascot server version 2.5 (Matrix Science, Boston, MA, USA)^65^ to identify the peptides and proteins in each sample. Our transcriptome assembly was used in the analysis with the following conditions: precursor mass tolerance; 6 ppm, production mass tolerance; 0.5 Da, enzyme; trypsin, max missed coverage; 2, fixed modification; carbamidomethylation at Cys, variable modification; *N*-acetylation at protein N-term and oxidation at Met, criteria for identification; *p* < 0.05 (MS/MS ion search). The protein amount was estimated based on the number of sequenced peptides per protein (emPAI)^51^.

### Bioinformatics analysis

All bioinformatics analyses were performed using Perl custom scripts with the G-language Genome Analysis Environment (v1.9.1)^66^. The statistical analyses and visualizations were implemented using the R package (v 3.2.1)^67^. The homology search was carried out with BLASTP. The sequence logo was generated by WebLogo 3^68^, and the sequence alignment figure was produced by MUSCLE^69^ and MView^70^. The signal peptide was predicted by SignalP (v 5.0)^71^ and PrediSi^72^, and the initiation codon in cDNA was predicted based on Kozak’s rule by ATGpr^73^.

## Supplementary information

Supplementary Information.

Supplementary Table S2

## Data Availability

Raw sequence reads used for genome assembly and expression analysis have been submitted to DDBJ SRA (sequence read archive). Accession numbers of transcriptome short reads are DRR155974 (*Octonoba yesoensis*), DRR156101 (*Octonoba sybotides*), DRR155866 (Octonoba okinawensis), DRR156045 (*Octonoba varians*), DRR157004 (*Octonoba grandiconcava*), and DRR157173 (*Octonoba grandiprojecta*), and nanopore long reads of genomic DNA are DRR238369 (*Octonoba okinawensis*), DRR238370 (*Octonoba varians*), DRR238371 (*Octonoba sybotides*). The assembled contigs are available at the DDBJ under the accession numbers IAQA01000001-IAQA01044186 (*Octonoba yesoensis*), IAUX01000001-IAUX01039949 (*Octonoba sybotides*), IALW01000001-IALW01038452 (*Octonoba okinawensis*), IAST01000001-IAST01041438 (*Octonoba varians*), ICDQ01000001-ICDQ01046746 (*Octonoba grandiconcava*), and ICKD01000001-ICKD01039972 (*Octonoba grandiprojecta*). Each spidroin sequence is available at the DDBJ under the accession number LC570186-LC570252.
