# Supplementary material for: Spidroin profiling of cribellate spiders provides insight into the evolution of spider prey capture strategies
Source: Sci Rep. 2020 Sep 24;10:15721. doi: 10.1038/s41598-020-72888-6 (PMC7515903; doi:10.1038/s41598-020-72888-6)
Supplement: Supplementary file 1 — Supplementary Information. [file 41598_2020_72888_MOESM1_ESM.docx]

**Supplemental Materials for**

Spidroin profiling of cribellate spiders provides insight into the evolution of spider prey capture strategies

Nobuaki Kono^1^*, Hiroyuki Nakamura^2^, Masaru Mori^1^, Masaru Tomita^1^, Kazuharu Arakawa^1^

1 Institute for Advanced Biosciences, Keio University, 246-2 Mizukami, Kakuganji, Tsuruoka, Yamagata, 997-0052, Japan

2 Spiber Inc., 234-1 Mizukami, Kakuganji, Tsuruoka, Yamagata 997-0052, Japan

* Corresponding author

**This Supplementary Material include:**

Figs S1 to S12, Tables S1 to S4

**Supplemental Figure S1**

Dotplot images of full-length spidroins (MiSp, MaSp2, CySp, and PySp in *O. sybotides*, and MiSp in *O. okinawensis*) obtained from direct DNA sequencing of gDNA. These spidroins have no intronic regions.

** Supplemental Figure S2**

Mapping results with Illumina short reads on full-length spidroins (Figure S1) and sequences. Continuous mapping results mean that these spidroin sequences are not the chimeric artifact.

**Supplemental Figure S3**

The structural information around the N-terminal region of CrSp in *O. sybotides*. Top graphs are the results of signal peptide prediction with SignalP and PrediSi. The asterisk marked M residues are start codon candidates based on the Kozak rule. Based on the above prediction results, the black arrow position is suitable as the start codon of CrSp.

**Supplemental Figure S4**

Alignment result of N-terminal region of CrSp in *O. sybotides* and *O. grandiprojecta*.

**Supplemental Figure S5**

The CrSp gene was obtained as three long reads with long-read sequencing of gDNA.

**Supplemental Figure S6**

The CrSp gene was obtained as three long reads with long-read sequencing of gDNA.

This graph represents an alignment result of *Pflag* genes obtained by this study and previous report^1^. Consensus patterns are based on conserved physicochemical classes^2^. Each lowercase letter and symbol (- or +) indicates the following meaning: o = alcohol {S, T}, l = aliphatic { I, L, V }, a = aromatic { F, H, W, Y }, c =  charged { D, E, H, K, R }, h = hydrophobic { A, C, F, G, H, I, K, L, M, R, T, V, W, Y }, - = negative { D, E }, p = polar { C, D, E, H, K, N, Q, R, S, T }, + = positive { H, K, R }, s = small { A, C, D, G, N, P, S, T, V }, u = tiny { A, G, S }, t = turnlike { A, C, D, E, G, H, K, N, Q, R, S, T }.

**Supplemental Figure S7**

This heatmap is constructed by the correlation coefficient of TPM scores among spidroins at Fig 2a.

**Supplemental Figure S8**

This boxplot represents the emPAI scores of each spidroin at whole web (Whole), radius and spidral area (Radius/Spiral), and stabilimentum (Stabilimentum) area (table S2).

**Supplemental Figure S9**

Phylogenic analysis of all spidroin N-terminal regions. Each spidroin type is coloured and Octonoba spiders are represented by bold font. Branch labels designate bootstrap support values. All spidroin data are listed in Tables S3 and S4.

**Supplemental Figure S10**

This graph represents an alignment result of MaSp genes obtained by this study and those previously reported. Consensus patterns are based on conserved physicochemical classes^2^. Each lowercase letter and symbol (- or +) indicates the following meaning: o = alcohol {S, T}, l = aliphatic { I, L, V }, a = aromatic { F, H, W, Y }, c =  charged { D, E, H, K, R }, h = hydrophobic { A, C, F, G, H, I, K, L, M, R, T, V, W, Y }, - = negative { D, E }, p = polar { C, D, E, H, K, N, Q, R, S, T }, + = positive { H, K, R }, s = small { A, C, D, G, N, P, S, T, V }, u = tiny { A, G, S }, t = turnlike { A, C, D, E, G, H, K, N, Q, R, S, T }.

**Supplemental Figure S11**

This graph represents an alignment result of CrSp repetitive domains obtained by this study and those previously reported. Consensus patterns are based on conserved physicochemical classes^2^. Each lowercase letter and symbol (- or +) indicates the following meaning: o = alcohol {S, T}, l = aliphatic { I, L, V }, a = aromatic { F, H, W, Y }, c =  charged { D, E, H, K, R }, h = hydrophobic { A, C, F, G, H, I, K, L, M, R, T, V, W, Y }, - = negative { D, E }, p = polar { C, D, E, H, K, N, Q, R, S, T }, + = positive { H, K, R }, s = small { A, C, D, G, N, P, S, T, V }, u = tiny { A, G, S }, t = turnlike { A, C, D, E, G, H, K, N, Q, R, S, T }.

**Supplemental Figure S12**

The protein structural features of CrSp (Octonoba yesoensis: this study) and AgSp and these figures are generated by NetSurfP-2.0^3^. The line graph represents the surface accessibility (red: exposed; blue: buried). The graphics mean the secondary structures. The orange wavy line is a helix, the purple arrow is a strand, and the pink line is a coil. The thickness of the gray line is a probability of disordered residue.

**Supplemental Table S1:** Summary statistics of long reads from gDNA sequencing

| **ONT gDNA sequence** | |  |  |  |  |
| --- | --- | --- | --- | --- | --- |
|  |  | ***Octonoba sybotides*** | ***Octonoba okinawensis*** | ***Octonoba varians*** | Average |
|  | Number of reads | 190,963 | 42,418 | 167,164 | 133,515 |
|  | Total length (kbp) | 2,673,686.77 | 551,814.12 | 2,341,686.43 | 1,855,729.11 |
|  | Average read length (kbp) | 14.00 | 13.01 | 14.01 | 13.67 |
|  | Longest read length (kbp) | 301.78 | 177.09 | 138.23 | 205.70 |

**Supplemental Table S2:** Result of mass spectrometry analysis at Fig. 2f.

SupplementalTableS2.xlsx

**Supplemental Table S3:** Summary of known spidroin genes used in the phylogenic analysis of C-terminal regions at Fig. 3a

| **C-terminal region** | | | | | |
| --- | --- | --- | --- | --- | --- |
|  | **Family** | ***Genus*** | ***species*** | **Spidroin** | **Accession number** |
|  | Uloboridae | *Uloborus* | *diversus* | AcSp | DQ399333 |
|  | Theridiidae | *Latrodectus* | *hesperus* | AcSp | JX978172 |
|  | Araneidae | *Argiope* | *trifasciata* | AcSp | AY426339 |
|  | Araneidae | *Araneus* | *ventricosus* | AcSp | AVEN_246635_1 |
|  | Araneidae | *Araneus* | *diadematus* | AgSp | AMK48674 |
|  | Nephilidae | *Trichonephila* | *clavipes* | AgSp | AMK48677 |
|  | Araneidae | *Araneus* | *ventricosus* | AgSp | AVEN_118932_1 |
|  | Zoropsidae | *Tengella* | *perfuga* | CrSp | Correa-Garhwal, et al. 2018 |
|  | Araneidae | *Argiope* | *aurantia* | CySp | AY953081 |
|  | Araneidae | *Araneus* | *ventricosus* | CySp | AVEN_18336_1 |
|  | Nephilidae | *Trichonephila* | *clavata* | CySp | AB218973 |
|  | Deinopidae | *Deinopis* | *spinosa* | CySp | AY953073 |
|  | Agelenidae | *Agelenopsis* | *aperta* | CySp | HM752572 |
|  | Theridiidae | *Latrodectus* | *hesperus* | CySp | DQ109035 |
|  | Araneidae | *Araneus* | *gemmoides* | CySp | AY855101 |
|  | Araneidae | *Cyrtophora* | *moluccensis* | CySp | AY953083 |
|  | Uloboridae | *Uloborus* | *diversus* | CySp | AY953072 |
|  | Nephilidae | *Trichonephila* | *inaurata* | Flag | AAF36092 |
|  | Nephilidae | *Trichonephila* | *clavipes* | Flag | AAC38847 |
|  | Nephilidae | *Nephilingis* | *cruentata* | Flag | ABR37273 |
|  | Araneidae | *Argiope* | *trifasciata* | Flag | AAK30593 |
|  | Araneidae | *Araneus* | *ventricosus* | Flag | AVEN_100465_1 |
|  | Araneidae | *Argiope* | *amoena* | MaSp2 | AY365018 |
|  | Nephilidae | *Nephila* | *pilipes* | MaSp | AY666054 |
|  | Araneidae | *Argiope* | *bruennichi* | MaSp2 | JX112872 |
|  | Uloboridae | *Octonoba* | *varians* | MaSp | AY666057 |
|  | Theridiidae | *Latrodectus* | *hesperus* | MaSp | EF595246 |
|  | Agelenidae | *Agelenopsis* | *aperta* | MaSp | AY566305 |
|  | Pisauridae | *Euprosthenops* | *australis* | MaSp | AM490178 |
|  | Theridiidae | *Latrodectus* | *geometricus* | MaSp | DQ059133S2 |
|  | Uloboridae | *Uloborus* | *diversus* | MaSp2 | DQ399334 |
|  | Araneidae | *Argiope* | *trifasciata* | MaSp2 | DQ059136S2 |
|  | Psechridae | *Psechrus* | *sinensis* | MaSp | AY666067 |
|  | Theridiidae | *Latrodectus* | *geometricus* | MaSp2 | AF350275 |
|  | Araneidae | *Argiope* | *bruennichi* | MaSp | JX112871 |
|  | Araneidae | *Argiope* | *trifasciata* | MaSp | AF350266 |
|  | Nephilidae | *Trichonephila* | *clavipes* | MaSp | AY654293 |
|  | Araneidae | *Araneus* | *ventricosus* | MaSp3 | AVEN_7867_1 |
|  | Araneidae | *Araneus* | *ventricosus* | MaSp1 | AVEN_121555_1 |
|  | Araneidae | *Araneus* | *ventricosus* | MaSp2B | AVEN_171746_1 |
|  | Nephilidae | *Trichonephila* | *senegalensis* | MaSp2 | AF350280 |
|  | Hexathelidae | *Macrothele* | *holsti* | MaSp | AY666068 |
|  | Pisauridae | *Euprosthenops* | *australis* | MaSp2 | AM490169 |
|  | Uloboridae | *Uloborus* | *diversus* | MaSp | DQ399331 |
|  | Deinopidae | *Deinopis* | *spinosa* | MaSp2 | DQ399329 |
|  | Araneidae | *Cyrtophora* | *moluccensis* | MaSp | KF032719 |
|  | Nephilidae | *Trichonephila* | *inaurata* | MaSp2 | AF350278 |
|  | Oxyopidae | *Peucetia* | *viridans* | MaSp | GU306168 |
|  | Tetragnathidae | *Tetragnatha* | *versicolor* | MaSp | AF350286 |
|  | Araneidae | *Araneus* | *ventricosus* | MaSp2A | AVEN_171745_1 |
|  | Araneidae | *Argiope* | *aurantia* | MaSp2 | AF350263 |
|  | Araneidae | *Gasteracantha* | *cancriformis* | MaSp2 | AF350272 |
|  | Theridiidae | *Latrodectus* | *hesperus* | MaSp2 | EF595245 |
|  | Tetragnathidae | *Tetragnatha* | *kauaiensis* | MaSp | AF350285 |
|  | Araneidae | *Metepeira* | *grandiosa* | MiSp | HM752569 |
|  | Araneidae | *Araneus* | *ventricosus* | MiSpB | AVEN_186744_1 |
|  | Theridiidae | *Latrodectus* | *hesperus* | MiSp | ACB29694 |
|  | Araneidae | *Argiope* | *argentata* | MiSp | JQ713004 |
|  | Araneidae | *Araneus* | *ventricosus* | MiSpA | AVEN_264011_1 |
|  | Nephilidae | *Trichonephila* | *clavipes* | MiSp | AAC14589 |
|  | Uloboridae | *Uloborus* | *diversus* | MiSp | DQ399332 |
|  | Araneidae | *Araneus* | *ventricosus* | PySp | AVEN_16081_1 |
|  | Theridiidae | *Latrodectus* | *hesperus* | PySp | FJ973621 |
|  | Araneidae | *Argiope* | *trifasciata* | PySp | GQ980328 |
|  | Eresidae | *Stegodyphus* | *mimosarum* | CrSp | KFM60634 |
|  | Nephilidae | *Trichonephila* | *clavipes* | PySp | GQ980330 |

**Supplemental Table S4:** Summary of known spidroin genes used in the phylogenic analysis of N-terminal regions at Fig. S9.

| **N-terminal region** | | | | | |
| --- | --- | --- | --- | --- | --- |
|  | **Family** | ***Genus*** | ***species*** | **Spidroin** | **Accession number** |
|  | Theridiidae | *Parasteatoda* | *tepidariorum* | AcSp | AWK58692 |
|  | Theridiidae | *Latrodectus* | *hesperus* | AcSp | JX978171 |
|  | Araneidae | *Araneus* | *diadematus* | AcSp | AWK58689 |
|  | Araneidae | *Araneus* | *ventricosus* | AcSp | AVEN_246635_1 |
|  | Araneidae | *Argiope* | *argentata* | AcSp | AWK58686 |
|  | Theridiidae | *Steatoda* | *grossa* | AcSp | AWK58693 |
|  | Araneidae | *Araneus* | *ventricosus* | AgSp | AVEN_118932_1 |
|  | Nephilidae | *Trichonephila* | *clavipes* | AgSp | PRD26655 |
|  | Theridiidae | *Steatoda* | *grossa* | AgSp | AWK58721 |
|  | Theridiidae | *Latrodectus* | *hesperus* | AgSp | AWK58720 |
|  | Araneidae | *Araneus* | *ventricosus* | CySp | AVEN_18336_1 |
|  | Agelenidae | *Agelenopsis* | *aperta* | CySp | HM752576-1 |
|  | Nephilidae | *Trichonephila* | *antipodiana* | CySp | EU730637 |
|  | Nephilidae | *Trichonephila* | *clavata* | CySp | AB218974 |
|  | Theridiidae | *Latrodectus* | *hesperus* | CySp | DQ379383 |
|  | Araneidae | *Araneus* | *diadematus* | Flag | AWK58735 |
|  | Araneidae | *Argiope* | *argentata* | Flag | AWK58732 |
|  | Araneidae | *Araneus* | *ventricosus* | Flag | AVEN_100465_1 |
|  | Araneidae | *Argiope* | *trifasciata* | MaSp2 | DQ059136S1 |
|  | Nephilidae | *Trichonephila* | *inaurata* | MaSp2 | DQ059135 |
|  | Araneidae | *Argiope* | *bruennichi* | MaSp2 | JX112872 |
|  | Araneidae | *Araneus* | *ventricosus* | MaSp3 | AVEN_7867_1 |
|  | Theridiidae | *Latrodectus* | *hesperus* | MaSp | EF595246 |
|  | Nephilidae | *Trichonephila* | *clavipes* | MaSp2 | EU599240 |
|  | Diguetidae | *Diguetia* | *canities* | MaSp | HM752564 |
|  | Araneidae | *Araneus* | *ventricosus* | MaSp1 | AVEN_121555_1 |
|  | Araneidae | *Araneus* | *ventricosus* | MaSp2A | AVEN_171745_1 |
|  | Araneidae | *Araneus* | *ventricosus* | MaSp2B | AVEN_171746_1 |
|  | Pisauridae | *Euprosthenops* | *australis* | MaSp | AM259067 |
|  | Theridiidae | *Latrodectus* | *geometricus* | MaSp | EU177668 |
|  | Agelenidae | *Agelenopsis* | *aperta* | MaSp | HM752573-1 |
|  | Theridiidae | *Latrodectus* | *hesperus* | MaSp2 | EF595245 |
|  | Araneidae | *Araneus* | *ventricosus* | MiSpB | AVEN_186744_1 |
|  | Theridiidae | *Latrodectus* | *hesperus* | MiSp | HM752570 |
|  | Theridiidae | *Steatoda* | *grossa* | MiSp | AWK58683 |
|  | Araneidae | *Araneus* | *ventricosus* | MiSpA | AVEN_264011_1 |
|  | Nephilidae | *Trichonephila* | *clavipes* | MiSp | AWK58680 |
|  | Araneidae | *Araneus* | *diadematus* | MiSp | AWK58673 |
|  | Araneidae | *Araneus* | *diadematus* | PySp | AWK58658 |
|  | Araneidae | *Araneus* | *ventricosus* | PySp | AVEN_16081_1 |
|  | Nephilidae | *Trichonephila* | *clavipes* | PySp | AWK58660 |
|  | Theridiidae | *Latrodectus* | *hesperus* | PySp | AWK58659 |
|  | Theridiidae | *Steatoda* | *grossa* | PySp | AWK58661 |
|  | Araneidae | *Argiope* | *argentata* | PySp | AQR58363 |

**References**

1 Garb, J. E., Dimauro, T., Vo, V. & Hayashi, C. Y. Silk genes support the single origin of orb webs. *Science* **312**, 1762 (2006).

2 Taylor, W. R. The classification of amino acid conservation. *J. Theor. Biol.* **119**, 205-218 (1986).

3 Klausen, M. S. *et al.* NetSurfP-2.0: Improved prediction of protein structural features by integrated deep learning. *Proteins* **87**, 520-527 (2019).
